# Design of Wideband Decoupling Antenna Pairs for 5G Portable Devices at N77/N78/N79 Bands

**DOI:** 10.3390/mi13111964

**Published:** 2022-11-12

**Authors:** Kaiwen Du, Yongshun Wang, Lijun Zhang, Yao Hu

**Affiliations:** 1 School of Electronic and Information Engineering, Lanzhou Jiaotong University, Lanzhou 730070, China; 2 Engineering Research Center of Integrated Circuit Packaging and Testing, The Ministry of National Education, Tianshui 741000, China; 3 School of Electronic Information and Electrical Engineering, Tianshui Normal University, Tianshui 741000, China; 4 Shool of Mechanical and Electrical Engineering, Lanzhou Jiaotong University, Lanzhou 730070, China

**Keywords:** decoupling antenna pairs, DGS, multiple-input multiple-output (MIMO), CMA

## Abstract

We proposed an antenna pair that is applicable for mobile terminals: a type of coupling feed planar antenna that has a size of $28\times 6{\;\mathrm{mm}}^{2}$, a wide band, coupling suppression and compact size. Different from the conventional antenna design, the process of band expansion comes from a dual-band antenna pair and is based on characteristic modes theory (CMA). By observing characteristic current distributions, the optimization emphasis is placed on current sensitive parts in antenna structures, which is an effective method to create or modify resonant points for exciting band potential. Meanwhile, the multiple defective ground structure (DGS) is introduced for isolation enhancement. The −10 dB bandwidth of 3.23–5.24 GHz can be realized, and the isolation of two antenna pairs with only 2 mm spacing is lower (−17.34 dB). The eight-port MIMO system constructed by four proposed antenna pairs has been fabricated. The simulated and measured results of MIMO indicate that its −10 dB operating bandwidth can work at N77/N78/N79/WLAN 5 GHz bands. Moreover, a lower ECC of less than 0.04 and a high efficiency of more than 60% can be obtained, which confirms that it is capable of excellent data transmission as a terminal MIMO antenna system.

## 1. Introduction

The process of wireless communications on mobile terminals needs to ensure the reliability and effectiveness of massive data transmissions. By transmitting data through several independent spatial channels, the multiple-input multiple-output (MIMO) antenna system can improve data transmission rates and increase channel capacity to maintain a well-communicated quality for mobile terminals. However, due to the limitation of device sizes and the increase of antenna numbers, mutual coupling exists inevitably in multiple antenna systems, which can reduce the antenna isolation and increase the radiation spatial correlation, thus indirectly decreasing the channel capacity and deteriorating the communication quality. Some methods of adding special structures for antenna decoupling are effective, such as neutralization lines (NLs) [1,2,3], parasitic elements (PEs) [4,5,6], lumped components [7,8,9], defective ground structures (DGSs) [10,11,12], metamaterials [13,14,15] and so on. Other methods that do not add decoupling structures can ensure signal independence by exciting modes with different polarization directions [16,17,18,19].

The planar bezel antennas are suitable for portable devices due to their portability, compactness and low-profile height. Table 1 shows the comparison of related mobile terminal antenna research. A planar-printed antenna design is proposed in [20]. The F-like monopole antenna facilitates the realization of wideband. Between the two antenna units with 10 mm spacing, NL is introduced for low frequency decoupling and two grounded antennas act as parasitic monopoles to reduce the high frequency coupling. The isolation lower than −15 dB can be obtained. A planar printed antenna consists of a driven strip monopole, and a parasitic shorted strip is proposed in [21]. The driven strip monopole and a parasitic shorted strip are excited to generate low-order and high-order resonant modes to form two wide operating bands centered at 830 and 2200 MHz. MIMO system designs in [22,23,24] integrated multiple single antennas. Although decoupling measures are not needed to ensure port independence, their structures were not compact and the impedance bandwidth was narrow. To establish more ports in limited space, Refs.[25,26,27] were constructed by antenna pairs. The inner distance within the antenna pair in [25] was only 1.2 mm. The grounding branch was introduced, which enabled isolation to enhance 10 dB, but the impedance bandwidth was just 5%. The antenna pairs with NLs achieved dual operating bands and good isolation below −15 dB [26], though NLs increased the antenna complexity and size. The T-shaped DGS and strip patches were added within the antenna pair in [27] to obtain a wide band of 57% and better isolation of −15 dB. To meet impedance matching, the parasitic patches occupied too much space. Thus, balancing wide bandwidth, high isolation and compact size when designing antenna pairs is challenging.

**Table 1:** Comparisons of MIMO terminal antenna designs.

Ref.	Antenna Pairs	Element Size $\left(mm^{2}\right)$	Decoupling Methods	Frequency (GHz)	Isolation (dB)	ECC	Total Efficiency (%)	Ports
[22]	-	6.5×6	-	3.4–3.6 (−10 dB)	<−10	<0.055	65–80	10
[23]	-	4.6×5.6	-	3.3–3.7 (−6 dB)	<−15	<0.1	50–75	8
[24]	-	14.9×7	-	3.4–3.6/4.8–5.0 (−10 dB)	<−16.5	<0.01	74–85/70–82	4
[25]	√	20×6.2	PEs	3.4–3.6 (−10 dB)	<−17	<0.1	-	4
[26]	√	49.5×6	NLs	2.3–2.4/3.3–3.6 (−6 dB)	<−15	<0.15	47–73	8
[27]	√	47×6	PEs and DGS	3.3–5.95 (−6 dB)	<−15	<0.11	47–78	8
**Pro.**	√	28×6	DGS	3.23–5.24 (−10 dB)	<−17.34	<0.04	50–89	8

In this study, a low-profile wideband antenna pair with high isolation and concise structure is designed. The −10 dB impedance bandwidth realizes 47% (3.23–5.24 GHz), and the inner distance within the antenna pair is only 2 mm. For coupling reduction, the C-shaped DGS with a length of 0.15 $\lambda_{0}$ ($\lambda_{0}$ is the free space wavelength at the center frequency) is designed, which enables a high isolation performance of no more than −17.34 dB in the whole working band. Compared to a conventional antenna pair, the proposed decoupling structure is suitable for wideband antenna and avoids increasing the element size. Characteristic modes analysis (CMA) is applied to guide band extension. The radiation characteristics of the proposed antenna pair and the related eight-port MIMO system are confirmed by simulation and measurement.

## 2. Analysis of Proposed Antenna Pair

### 2.1. Configuration

**Figure 1:**
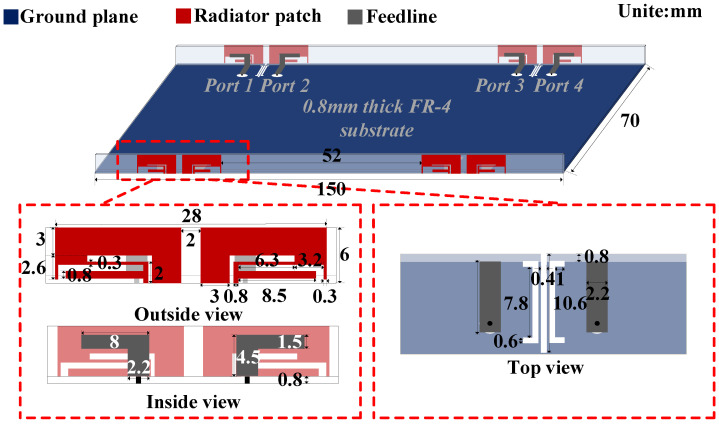
Configurations of the proposed antenna pair.

The proposed decoupling antenna pair with the dimension of $28\times 6{\;\mathrm{mm}}^{2}$ consists of two symmetrical antenna elements, as detailed in Figure 1. It is etched in an FR-4 substrate, which has a relative permittivity of 4.3, relative permeability of 1 and loss tangent of 0.0009. Each antenna element is formed by two radiating parts. One is an $\;8\times 4.5{\;\mathrm{mm}}^{2}$ L-shaped patch on the inside of the border acting as a feeding line and primary resonant part; the other is a $\;13\times 6{\;\mathrm{mm}}^{2}$ composite shape patch on the outside of the border to expand bandwidth and serves as the secondary resonant part. The internal spacing is only $0.03\lambda_{0}$. To overcome the severe mutual coupling caused by this compact arrangement, a C-shaped DGS etched on the ground plane is added in the middle of the symmetrical antenna elements to realize isolation enhancement and improve impedance matching further.

### 2.2. Antenna Pair Evolution

**Figure 2:**
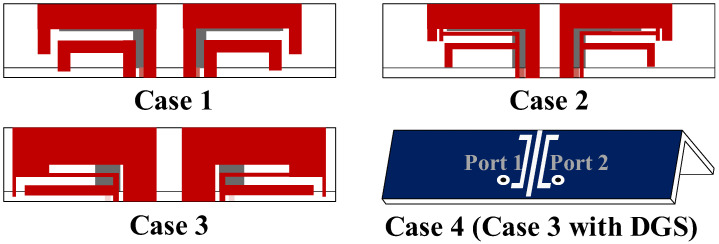
Design evolution of the proposed antenna pair.

**Figure 3:**
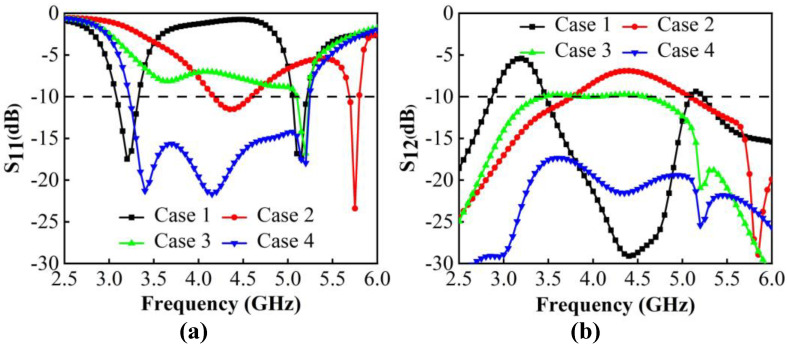
Evolution of S-parameters. (**a**) Reflection coefficients. (**b**) Transmission coefficients.

The realization of wideband is attributed to exciting multiple resonant points in the same plane, which is attained by designing resonant patches with different sizes. A feeding method that couples inner patches to feed outer patches has been chosen due to its ability to increase the equivalent capacitance, which also leads to the potential for wideband. To better understand the structure and operating principle, the process of obtaining the ideal antenna pairs is displayed in Figure 2. For band extension, CMA is adopted to capture more resonant modes in the desired bands. The modal significance (MS) shows the potential modes that can be excited in such structures. The modal weighting coefficient (MWC) reveals the resonance power of each mode when adding excitation sources. Due to the symmetry, some simulations are performed with port 1 being excited. The simulation results of the reflection coefficients and transmission coefficients are given in Figure 3.

**Figure 4:**
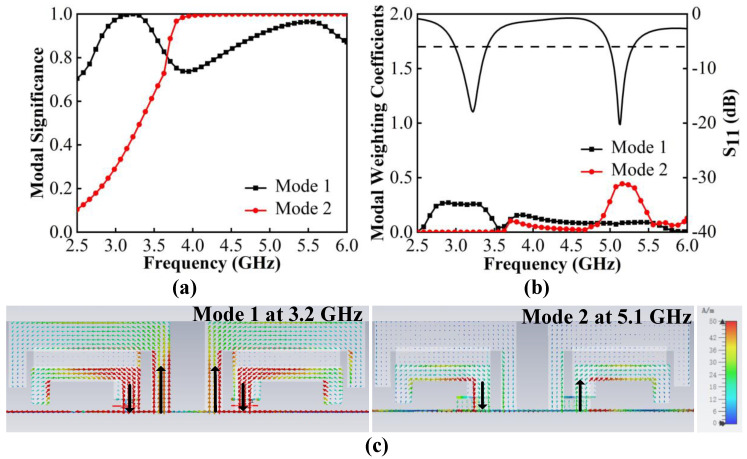
CMA of Case 1. (**a**) MS. (**b**) MWC. (**c**) Characteristic current distributions.

We begin by constructing a dual-band antenna with two L-shaped patches (Figure 2. Case 1). Figure 4a,b shows that two modes are significant and can be excited at 3.2 GHz and 5.1 GHz. Figure 4c shows that the characteristic currents of Mode 1 have a loop path on the two L-shaped patches, and Mode 2 concentrates on the short L-shaped patch only. To create possibilities for more resonant bands, the part of outer patches with apparent currents is modified to increase the number of resonant modes.

**Figure 5:**
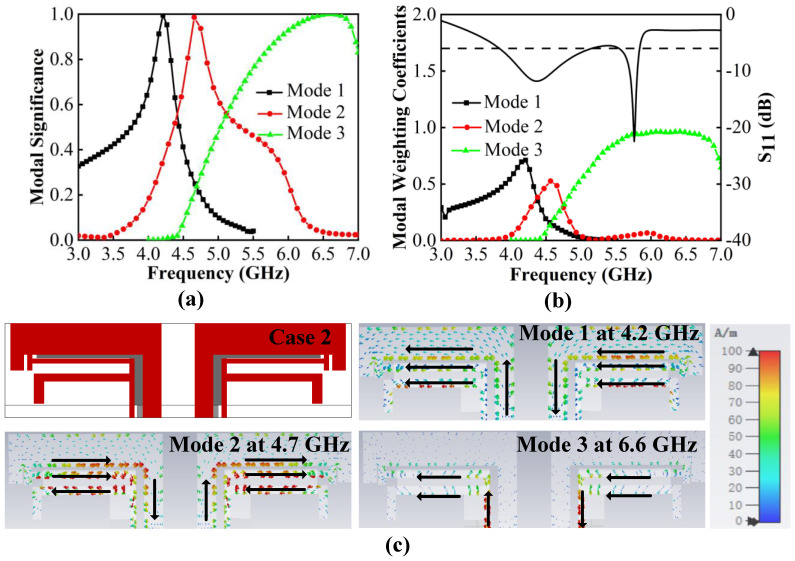
CMA of Case 2. (**a**) MS. (**b**) MWC. (**c**) Characteristic current distributions.

Thus, we tried to add a T-shaped patch to the middle of two L-patches. After optimizing, Case 2 is obtained. It can be seen from Figure 5a that three new characteristic modes emerge with resonance at 4.2 GHz, 4.7 GHz and 6.6 GHz. Figure 5b demonstrates that these modes can be effectively excited using a common exciting source, which works together to cause Case 2 to have two minimum resonance values at 3.8–5.8 GHz. The characteristic currents in Figure 5c illustrate that Mode 1 has an in-phase path on two L-patches and one T-patch; among them, the two L-patches are strong and the T-patch is weak. Mode 2 is strong on both patches and appears in an out-phase path on the T-patch and short L-patch. Mode 3 is resonated by currents only concentrated on T-patch and short L-patch. The introduction of the T-patch greatly contributes to the creation of more modes, but this is not enough. Furthermore, the resonant points need to be shifted toward the low frequency in order to reduce the resonant frequency. The length of the antenna pairs is increased when extending characteristic current paths. Moreover, to improve the impedance matching of Mode 2, the optimization is focused on the T-patch and the short L-patch, which are sensitive to mode currents. Next, Case 3 is obtained.

**Figure 6:**
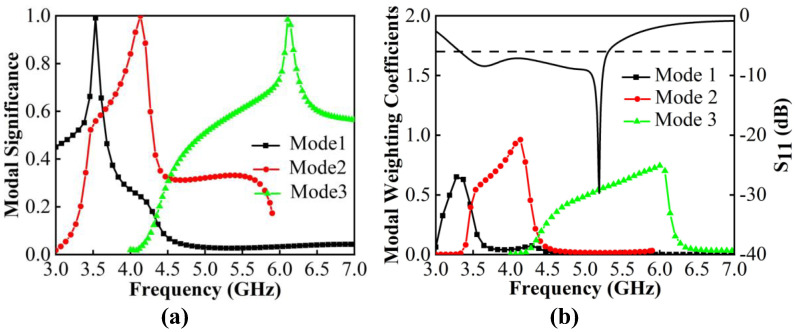
CMA of Case 3. (**a**) MS. (**b**) MWC.

Figure 6a shows that the resonant points of the original modes shift to the left on 3.6 GHz, 4.2 GHz and 6.1 GHz. Moreover, Mode 2 is greatly affected. Not only is the resonant point changed, but the mode bandwidth is also wider, which facilitates it to be significantly excited. Figure 6b shows that when feeding sources are added, all modes can be successfully resonated, and Mode 2 greatly supports and improves 3.3–5.3 GHz resonances of −6 dB in Case 3.

**Figure 7:**
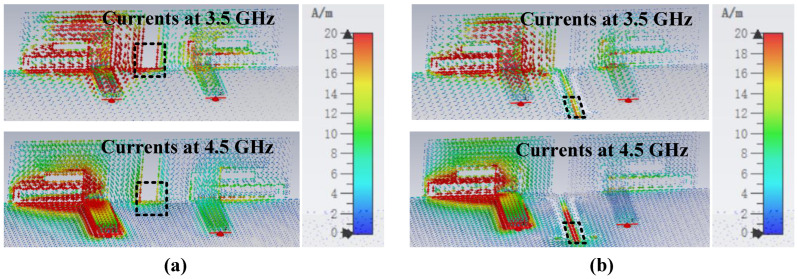
Comparisons of current distributions. (**a**) Case 3. (**b**) Case 4.

**Figure 8:**
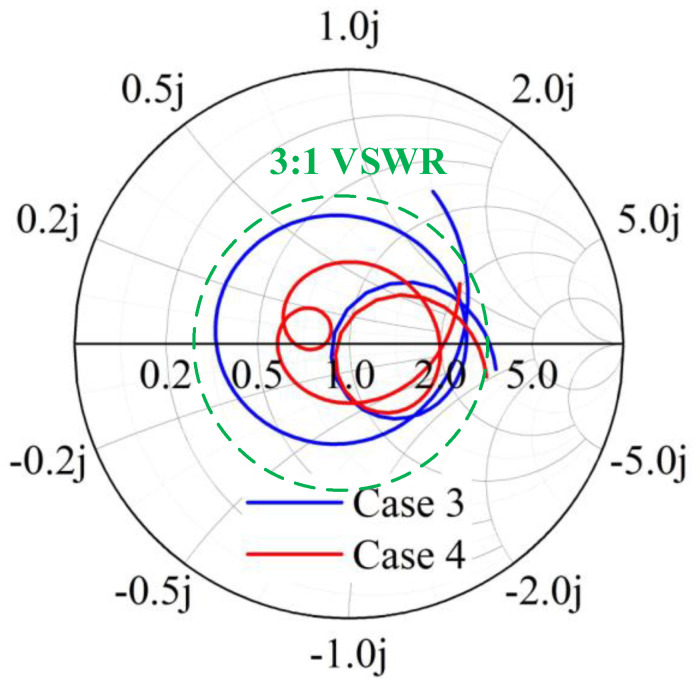
Comparisons of Case 3 and Case 4. Smith charts from 3.2–5.3 GHz.

The decoupling structure needs to be effective over a wide frequency band, which is challenging in comparison to conventional decoupling methods. Inspired by the wideband decoupling method using multiple NLs [28] and the multiple DGS decoupling method [29], multiple DGS with a length of 0.15 $\lambda_{0}$ are inserted between two antenna elements for isolation enhancement (Case 4). The S-parameters from Figure 3b indicate that the isolation of Case 3 is just less than −9.5 dB. The currents in Figure 7a illustrate that the poor isolation, which mainly exists on 3.5–4.5 GHz, is due to the surface waves on the common ground plane. It is evident from Figure 7b that when Port 1 is excited, the resonated surface currents fall into the proposed DGS rather than directly flow to the unexciting antennas, which blocks the surface currents propagation. Correspondingly, the coupling is well suppressed and an apparent reduction of approximately 7.8 dB of isolation is realized. Moreover, the bandwidth is changed. The Smith chart in Figure 8 analyzes the improvement of matching caused by decoupling structures from the variation of equivalent impedance. It is apparent that under the impact of DGS, the input impedance locations move clockwise and get closer to the matching point; such decoupling structures are the same as adding a parallel capacitor and a series inductor to the antenna pair. The lower the frequency, the more significant the effect of its capacitive reactance. The final proposed antenna pairs (Case 4) can achieve wide bandwidth of 3.23 GHz–5.24 GHz in −10 dB and good isolation of less than −17.34 dB.

### 2.3. Parameters Analysis

**Figure 9:**
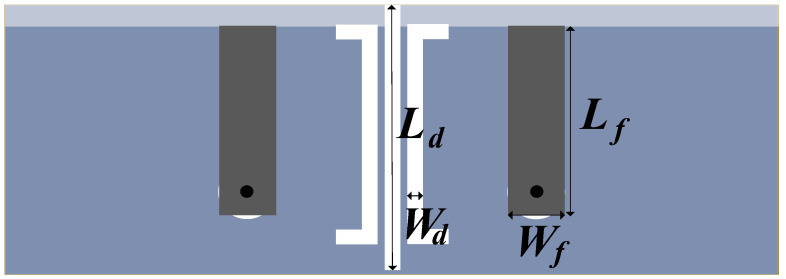
Variable position.

In the following analysis, the contribution by DGS and the feedline in the antenna pair work is discussed through the analysis of key parameters. The variables are shown in Figure 9. The length $L_{f}$ and width $W_{f}$ of the feedline are selected as variables. The length $L_{d}$ and width $W_{d}$ of the DGS are chosen as variables to be analyzed, where the three rectangular DGSs have the same width and the length is a proportionally related change.

**Figure 10:**
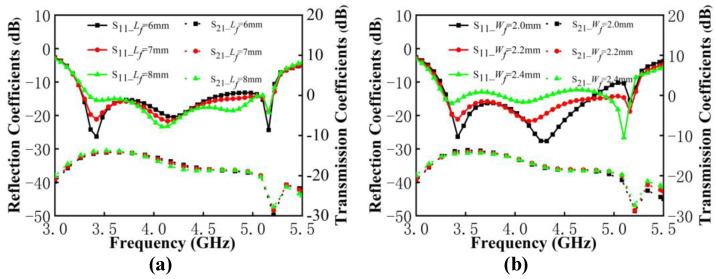
(**a**) S-parameters of proposed antenna pairs with different values of $L_{f}$. (**b**) S-parameters of proposed antenna pairs with different values of $W_{f}$.

It can be observed from Figure 10 that the value variation of the feedline size determines the impedance matching and has almost no effect on the isolation of antenna pairs. In Figure 10a, as the length of the feedline increases, the resonance point does not move. The impendence matching at low frequency is significantly worse and is slightly improved at high frequency. In Figure 10b, when the width increases, the resonant modes shift to the low frequency, and the matching situation at low frequency and high frequency exhibits a significant opposite trend. Therefore, in order to meet the good impedance matching in a wide frequency band, the reflection coefficient performance of both low and high frequencies should be considered. The length of 7 mm and the width of 2.2 mm on the feedline have been selected.

**Figure 11:**
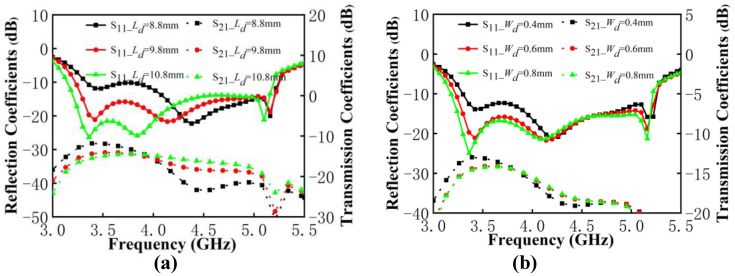
(**a**) S-parameters of the proposed antenna pairs with different values of $L_{d}$. (**b**) S-parameters of the proposed antenna pairs with different values of $W_{d}$.

As shown in Figure 11a, when the $L_{d}$ increases, the resonant frequency moves to the left. The impendence matching at the low frequency improved significantly, but is deteriorated a little at the high frequency. The isolation within the antenna pairs is greatly affected by opposite changes in low and high frequencies, such as when the isolation below 3.9 dB decreases and the isolation above 3.9 dB deteriorates. It is notable that the improvement in isolation by DGS becomes minimal when the value of $L_{d}$ exceeds 9.8 mm. In Figure 11b, the matching at the low frequency is affected by the value of $W_{d}$. When the value of $W_{d}$ exceeds 0.6 mm, it will have an insignificant effect on the isolation of the antenna pair. After the aforementioned discussion, the decoupling structure chose DGS with 9.8 mm in length and 0.6 mm in width.

## 3. Performances of the Eight-Port MIMO Antenna System

### 3.1. Simulated and Measured Results for MIMO System in Free Space

#### 3.1.1. S-Parameters, Total Efficiency and Peak Gain

**Figure 12:**
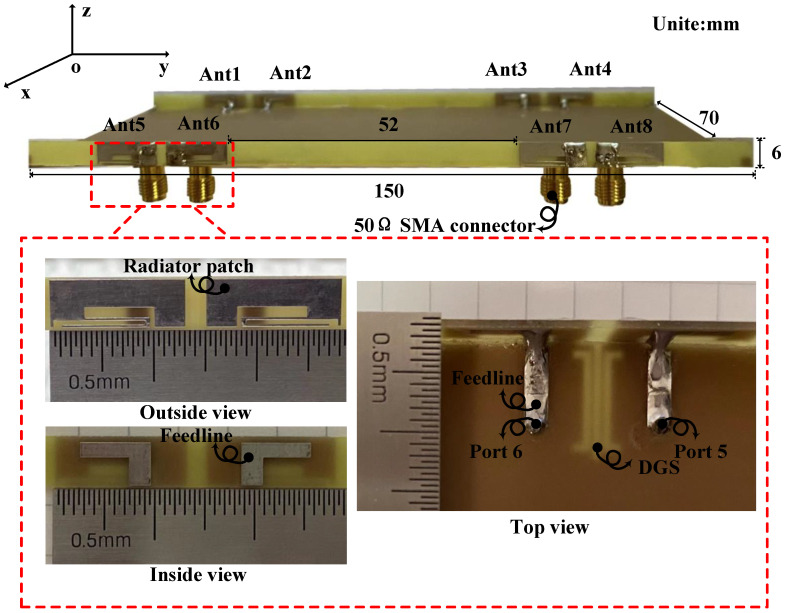
Geometry and photos of the fabricated eight-port MIMO system.

The four antenna pairs mentioned above are arranged on both sides of the $\;150\times 6\times 0.8{\;\mathrm{mm}}^{3}$ bezel to configure the eight-port MIMO system as detailed in Figure 12. The edge-to-edge distance between Ant2 and Ant3 is 52 mm and the remaining part can be left for placing other antennas as 3G/4G or WiFi modules. Eight SMA connectors are placed on the back of the substrate for antenna port testing. Due to the symmetrical arrangement of the MIMO system, similar results are no longer displayed.

**Figure 13:**
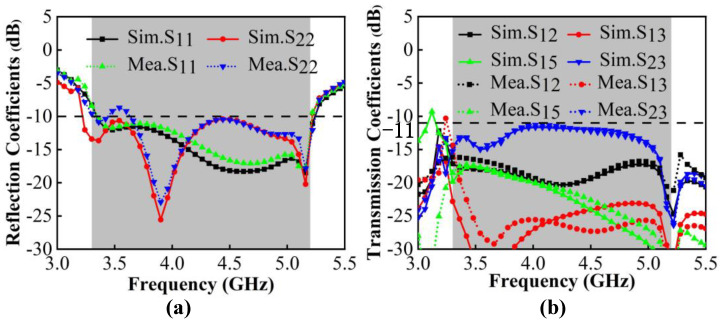
(**a**) Simulated and measured reflection coefficients. (**b**) Simulated and measured transmission coefficients.

According to the simulation and measurement results in Figure 13, there are slight differences in the S-parameters between them due to the addition of the SMA connectors and the unavoidable measurement errors. The operating frequency in −10 dB is 3.30 GHz–5.23 GHz with a bandwidth length of 1930 MHz; the target bands, N77/N78/N79, that can be fully covered and the WLAN 5 GHz band are partially included. The worst isolation excites in Ant2 and Ant3, which is lower than −11 dB.

**Figure 14:**
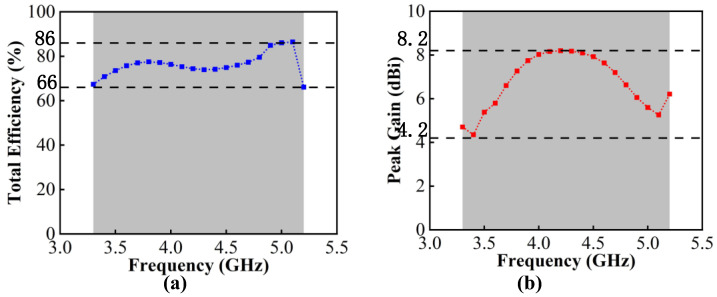
(**a**) Measured total efficiency. (**b**) Measured peak gain.

The total efficiency reflects the loss power and the radiated power situation of the antenna, which is the parameter affected by mutual coupling. It can be observed from Figure 14a that the measured total efficiency of the proposed MIMO systems ranges from 66% to 86%, which can meet the requirements for the total efficiency of more than 50% for the internal mobile terminal antenna. The measured gain is shown in Figure 14b. The value ranges from 4.2 dBi to 8.2 dBi.

#### 3.1.2. Diversity Performance

To further investigate the independence of each port, the envelope correlation coefficients (ECC) of the antenna elements are used to evaluate the spatial irrelevance of the antenna radiation. The ECC can be approximately calculated from the S-parameter, which is given in Equation (1).

(1) $$\mathrm{ECC}\left(m,n\right)=\frac{{\left|S_{mm}^{*}S_{mn}+S_{nm}^{*}S_{nn}\right|}^{2}}{\left\{1-\left({\left|S_{mm}\right|}^{2}+{\left|S_{nm}\right|}^{2}\right)\right\}\left\{1-\left({\left|S_{nn}\right|}^{2}+{\left|S_{mn}\right|}^{2}\right)\right\}}$$

**Figure 15:**
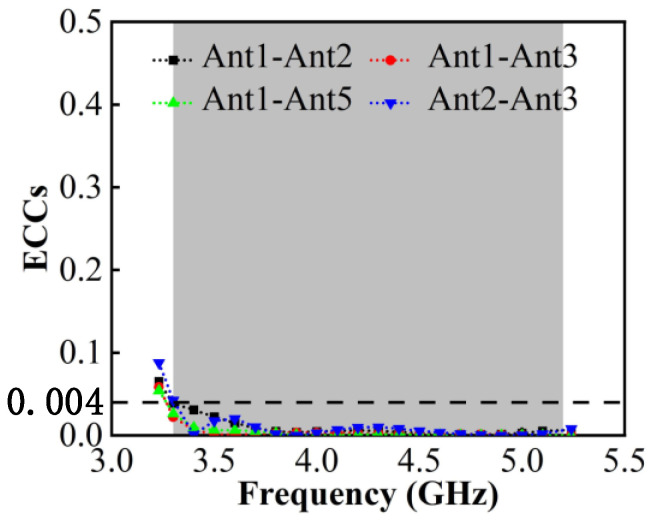
Calculated ECCs.

Figure 15 shows that the value of the calculated ECC is below 0.04 in the entire band and that in 3.8–5.0 GHz, it can be well below 0.01, which proves that the proposed MIMO has a good mutual coupling suppression ability.

The diversity gain (DG) reflects the ability to differentiate independent multipath signals on MIMO antenna systems, which can be calculated from ECCs in Equation (2).

(2) $$\mathrm{DG}\left(m,n\right)=10\sqrt{1-\mathrm{ECC}{\left(m,n\right)}^{2}}$$

**Figure 16:**
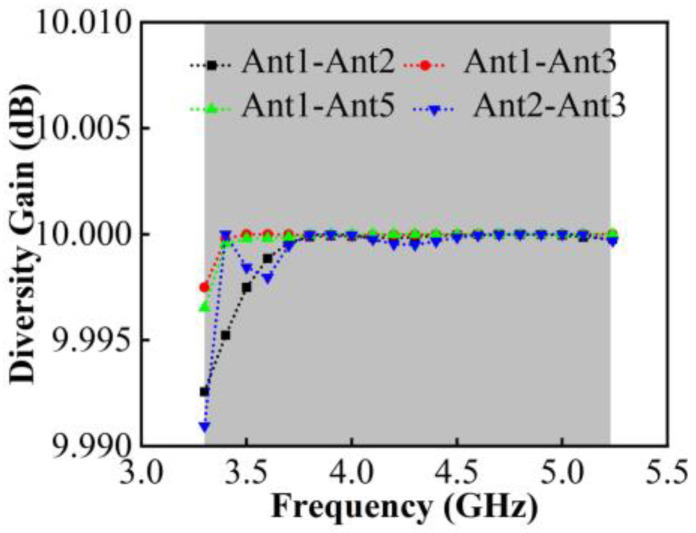
Calculated DG.

It can be observed in Figure 16 that in 3.30–5.23 GHz working bands, the value of DG is better than 9.99 dB.

**Figure 17:**
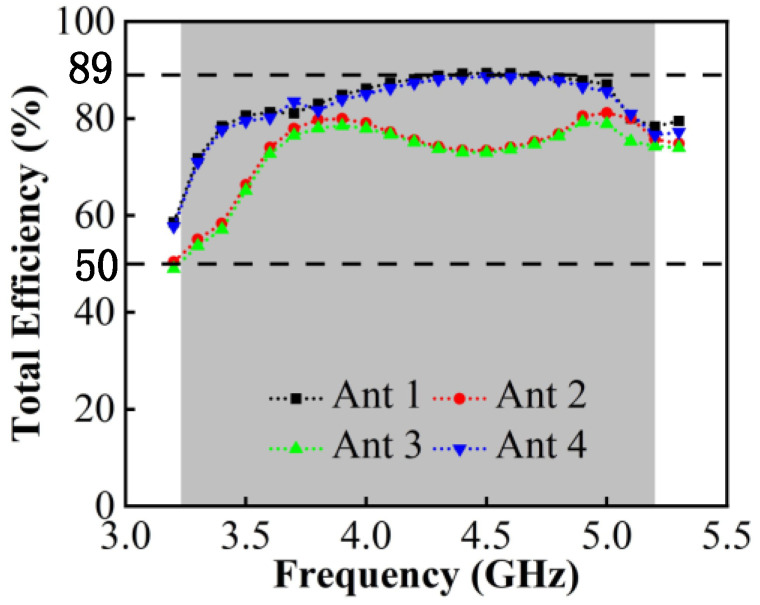
Measured total efficiency.

Figure 17 indicates the total efficiency of single antennas, which is above 50% and can reach up to 89%. From there, the MIMO antenna system carries spatial multiplexing and diversity techniques to improve the transmission rate, and its ports multiplexing efficiency can be calculated by Equation (3).

(3) $$\eta_{mux}\left(m,n\right)=\sqrt{\eta_{m}\eta_{n}\left(1-\left|\rho_{mn}{}^{2}\right|\right)}$$

**Figure 18:**
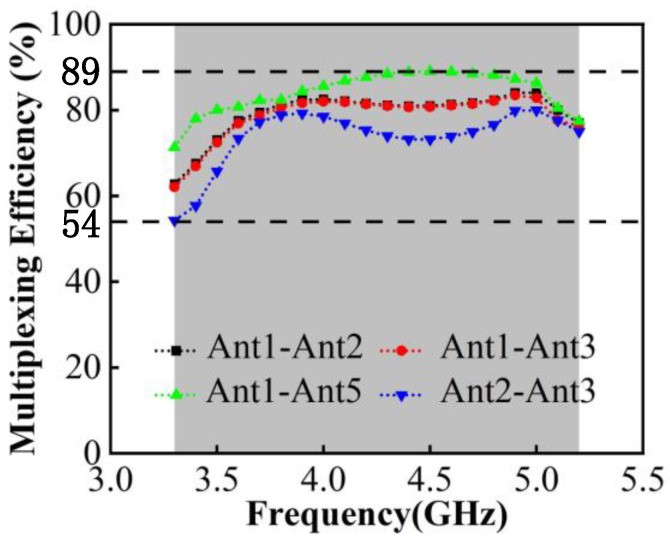
Calculated Multiplexing efficiency.

Among them, $\eta_{m}$ is the total efficiency of port m, $\eta_{n}$ is the total efficiency of port n and $\rho_{mn}$ is the correlation coefficient of the two ports. The above measurements are brought into Equation (2) and the calculation results are shown in Figure 18. It can be seen that the worst multiplexing efficiency occurs between antenna 2 and 3, which is due to the spatial correlation caused by the crossed radiation direction of the two antenna elements, but is still higher than 54%. The highest multiplexing efficiency occurs between antenna 1 and 5, which can reach 89%.

#### 3.1.3. Radiation Pattern

**Figure 19:**
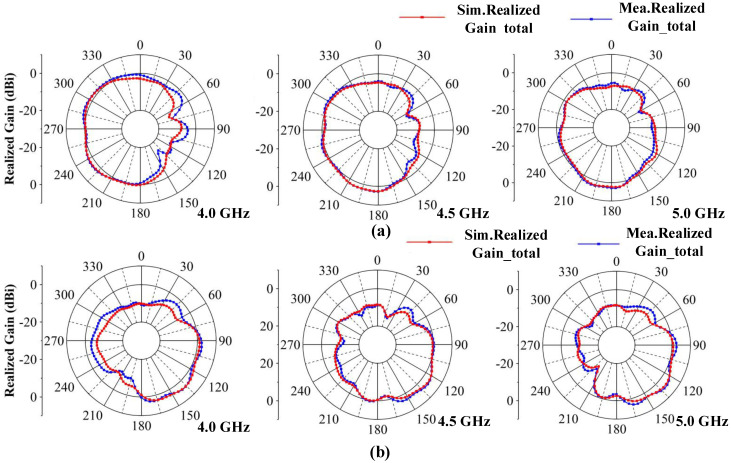
Simulated and measured radiation patterns of the proposed MIMO system in the xoy plane. (**a**) Ant 1. (**b**) Ant 2.

**Figure 20:**
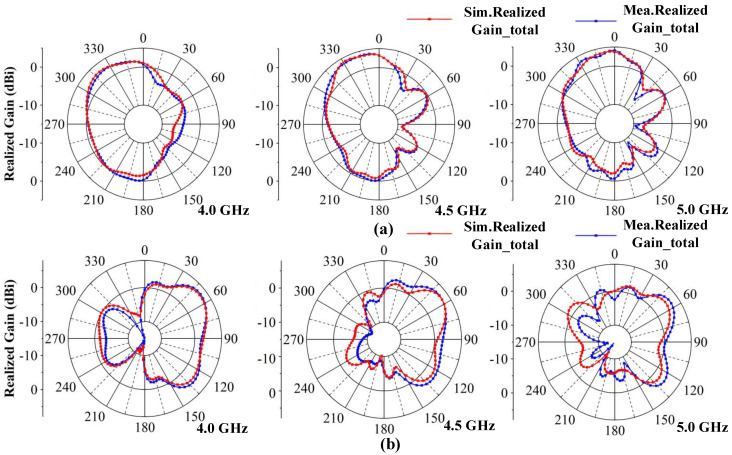
Simulated and measured radiation patterns of the proposed MIMO system in the yoz plane. (**a**) Ant 1. (**b**) Ant 2.

The two-dimensional (2D) measured radiation patterns are observed from three resonance points at 4.0, 4.5 and 5.0 GHz. Due to symmetry, only the results for Ant 1 and Ant 2 are shown. Figure 19 illustrates the radiation patterns in the xoy plane and Figure 20 illustrates the radiation patterns in the yoz plane. It can be seen that there is good agreement between the simulation results and measurement results. As the frequency shifts to higher frequencies, the maximum gain of the antennas increases, and the shapes of the radiation patterns have some variations at different frequencies, which are caused by different dominant resonant modes. The maximum radiation directions of the antennas are complementary, which ensures that the proposed MIMO system provides omnidirectional radiation patterns and good radiation independence of the different ports.

### 3.2. Discussion of Proposed MIMO Antenna System Performance in Practical Applications

#### 3.2.1. Handheld Effects

**Figure 21:**
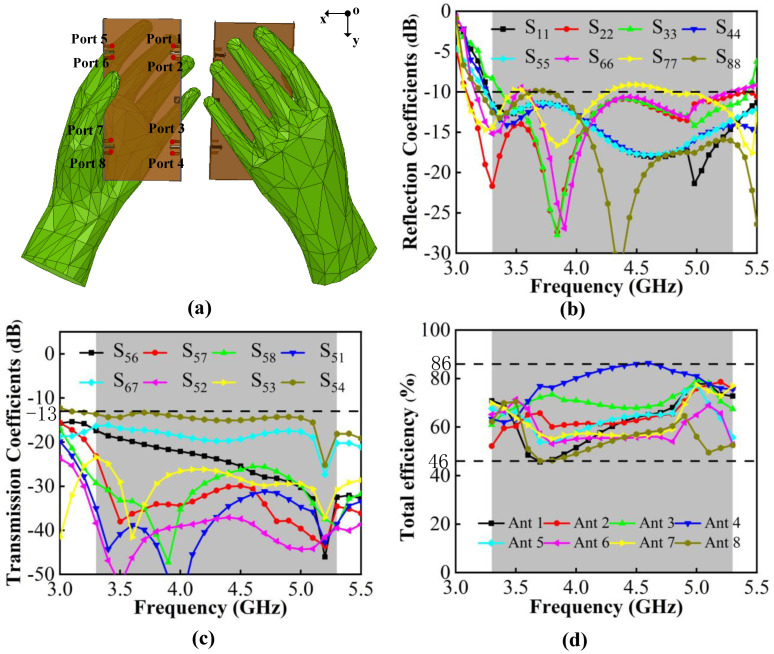
(**a**) MIMO system with user’s left hand model. (**b**) Simulated reflection coefficients. (**c**) Simulated transmission coefficients. (**d**) Simulated total efficiency.

**Figure 22:**
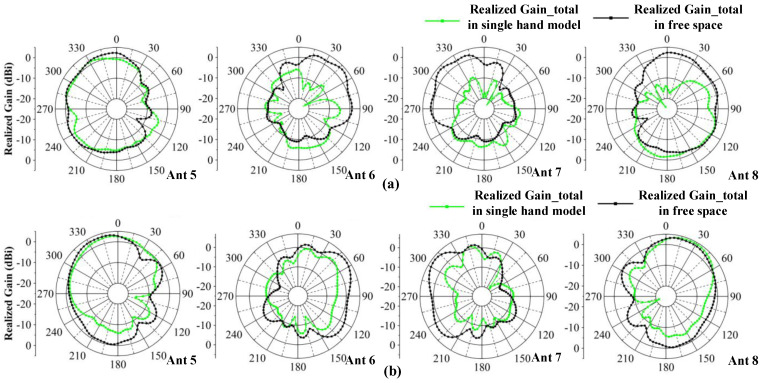
Comparisons of radiation pattern observed from 4.5 GHz. (**a**) 2-D radiation pattern in the xoy plane. (**b**) 2-D radiation pattern in in the yoz plane.

To verify the robustness of the proposed MIMO antenna system, the user’s hand effect is investigated. Figure 21a shows the MIMO system with user’s left hand, which simulates users talking on the phone or browsing messages. Ant 5–8 are close to the palm of the hand and Ant 1–4 are located in an open position. Figure 21b,c shows that S-parameters are less affected by the hand, which also can cover N77/N78/N79 bands, and that the worst isolation can lower than −13 dB. The total efficiency shown in Figure 16 illustrates that, in the case of a single handheld, the efficiency is impacted slightly and its value is reduced to 40–76%. The comparison of radiation patterns in the condition of single handheld and free space is also given in Figure 22. The observation frequency is 4.5 GHz and the results of Ant 5–8 are shown. One can observe that the radiation of Ant 6 and Ant 7 are most affected by the hand and have reduction in the realized gain. Ant 5 and Ant 8 can maintain the characteristics in the free space.

**Figure 23:**
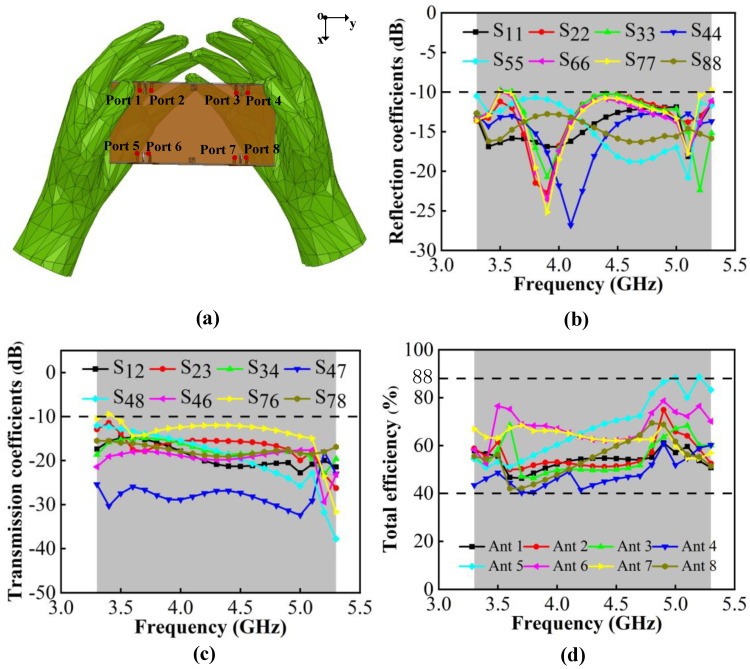
(**a**) MIMO system with user’s double-handed model. (**b**) Simulated reflection coefficients. (**c**) Simulated transmission coefficients. (**d**) Simulated total efficiency.

**Figure 24:**
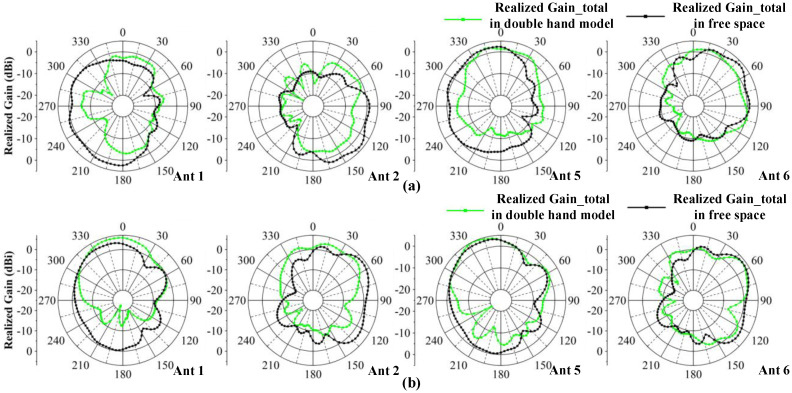
Comparisons of the radiation pattern observed from 4.5 GHz. (**a**) 2-D radiation pattern in the xoy plane. (**b**) 2-D radiation pattern in in the yoz plane.

Figure 21a shows the model simulating the antenna held in both hands, which imitates users playing games or watching videos. One can see from Figure 23b,c that in this situation, the N77/N78/N79 working bands can be kept. The worst isolation exists in Ant 6 and Ant 7, which are lower than −10 dB. The isolation within the antenna pairs can be lower than −15 dB. The total efficiency is from 40% to 88%. Among the antennas, Ant 1, Ant 2, Ant3 and Ant4 are influenced most by the user’s hand. Figure 24 shows the comparison of proposed antenna radiation patterns. The results show that Ant 1 and Ant 2 are more disturbed by hand interference, and that Ant 5 and Ant 6 have small differences compared to the case of free space. In conclusion, compared with the situation of free space, the model can maintain good port independence and radiation properties under the user’s hands.

#### 3.2.2. Mobile Phone Model Effects

**Figure 25:**
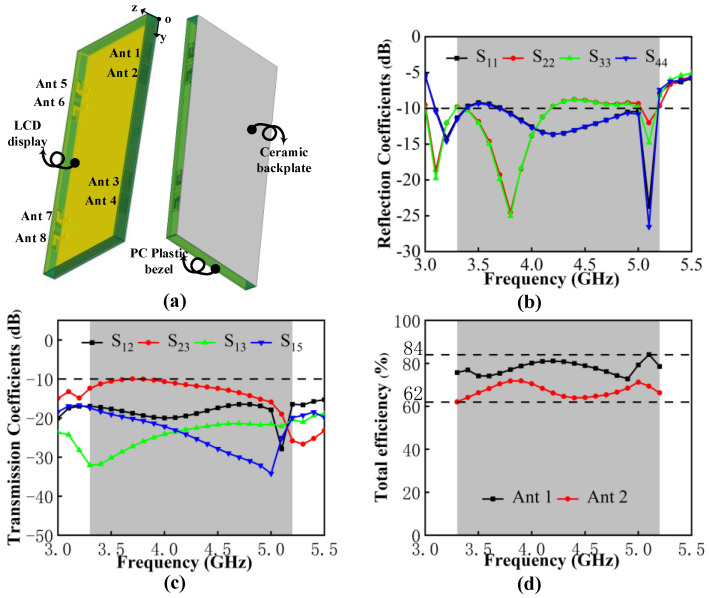
(**a**) MIMO system with mobile phone model. (**b**) Simulated reflection coefficients. (**c**) Simulated transmission coefficients. (**d**) Simulated total efficiency.

**Figure 26:**
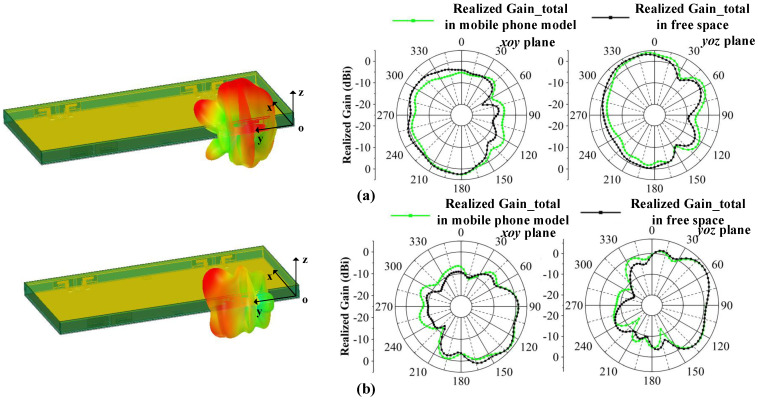
Radiation pattern of MIMO system with mobile phone model observed from 4.5 GHz. (**a**) 3-D and 2-D radiation pattern of Ant 1. (**b**) 3-D and 2-D radiation pattern of Ant 2.

The influences of LCD display (ε = 4.8), PC plastic bezel (ε = 3.13) and zirconia ceramic backplate (ε = 26) are presented in Figure 25. Compared to the MIMO system in free space, the working band shifts slightly to a lower band and the −10 dB impendence matching is minorly damaged in 3.5 GHz and 4.2–5.0 GHz. The isolation can be kept lower than −10 dB and the total efficiency performance remains satisfactory, from 62–84%. Figure 26 illustrates how the radiation patterns after adding the mobile phone model is almost unchanged, which means that the proposed MIMO antenna system is suitable for mobile terminal applications.

## 4. Conclusions

This paper proposes a design of an antenna pair with wide frequency bands, compact structure and coupling suppression. Compared to the traditional method of satisfying antenna matching through trial and error, the process of bandwidth expansion in this design is from the resonant mode perspective based on characteristic mode analysis. The simulated results show that the proposed antenna pair can achieve 47% (3.23–5.24 GHz) in −10 dB impedance bandwidth and lower than −17.34 dB isolation by adopting the multiple DGS decoupling method. In addition, an eight-port MIMO antenna system containing such antenna pairs is built. The MIMO system performance is simulated and measured and the results show a −10 dB bandwidth from 3.30–5.23 GHz, isolation below −11 dB, ECC below 0.04, total efficiency above 66%, gain above 4.2 dB and good diversity characteristics. In addition, the impact of a handheld and mobile phone model on this MIMO system is discussed, and it is further demonstrated that the proposed MIMO antenna system has potential for future mobile terminal applications.

## Data Availability

Not applicable.
